# Deubiquitinase MYSM1 in the Hematopoietic System and beyond: A Current Review

**DOI:** 10.3390/ijms21083007

**Published:** 2020-04-24

**Authors:** Amanda Fiore, Yue Liang, Yun Hsiao Lin, Jacky Tung, HanChen Wang, David Langlais, Anastasia Nijnik

**Affiliations:** 1 Department of Physiology, McGill University, Montreal, QC 3655, Canada; amanda.fiore@mail.mcgill.ca (A.F.); yue.liang@mail.mcgill.ca (Y.L.); yun.h.lin@mail.mcgill.ca (Y.H.L.); lin.tung@mail.mcgill.ca (J.T.); han.c.wang@mail.mcgill.ca (H.W.); 2 Research Centre on Complex Traits, McGill University, Montreal, QC 3649, Canada; david.langlais@mcgill.ca; 3 Department of Human Genetics, McGill University, Montreal, QC 3640, Canada; 4 McGill University Genome Centre, Montreal, QC 740, Canada

**Keywords:** deubiquitinase, hematopoiesis, hematopoietic stem cells, immune response, regulation of gene expression

## Abstract

MYSM1 has emerged as an important regulator of hematopoietic stem cell function, blood cell production, immune response, and other aspects of mammalian physiology. It is a metalloprotease family protein with deubiquitinase catalytic activity, as well as SANT and SWIRM domains. MYSM1 normally localizes to the nucleus, where it can interact with chromatin and regulate gene expression, through deubiquitination of histone H2A and non-catalytic contacts with other transcriptional regulators. A cytosolic form of MYSM1 protein was also recently described and demonstrated to regulate signal transduction pathways of innate immunity, by promoting the deubiquitination of TRAF3, TRAF6, and RIP2. In this work we review the current knowledge on the molecular mechanisms of action of MYSM1 protein in transcriptional regulation, signal transduction, and potentially other cellular processes. The functions of MYSM1 in different cell types and aspects of mammalian physiology are also reviewed, highlighting the key checkpoints in hematopoiesis, immunity, and beyond regulated by MYSM1. Importantly, mutations in MYSM1 in human were recently linked to a rare hereditary disorder characterized by leukopenia, anemia, and other hematopoietic and developmental abnormalities. Our growing knowledge of MYSM1 functions and mechanisms of actions sheds important insights into its role in mammalian physiology and the etiology of the MYSM1-deficiency disorder in human.

## 1. Introduction

### 1.1. Overview of MYSM1 Protein Structure and Catalytic Activity

In recent years, Myb-like, SWIRM, and MPN domains 1 (MYSM1) has emerged as an essential regulator of hematopoiesis, immunity, and other aspects of mammalian physiology. It is primarily a nuclear chromatin-interacting protein, with orthologues found only in the vertebrate species, indicating more recent evolutionary origins and suggesting specialized biological functions.

**Figure 1:**
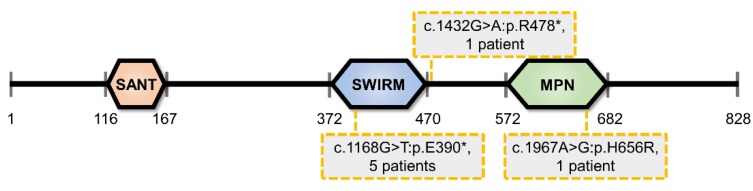
MYSM1 Protein: Domain Structure and Mutations in Human Patients. MYSM1 comprises SANT, SWIRM, and MPN domains. MYSM1 mutations reported in human MYSM1-deficiency syndrome patients include the p.H656R substitution within the critical JAMM-motif of the catalytic domain, and the nonsense variants p.E390* and p.Arg478* truncating MYSM1 protein upstream of the catalytic domain. All mutations in the patients are in a homozygous state.

Structurally MYSM1 comprises SANT, SWIRM, and MPN domains (Figure 1). The SANT domain of MYSM1 is structurally similar to the DNA-binding domain of transcription factor cMYB [1,2]. It can bind to DNA in vitro [2] and is required for MYSM1 association with histones in vivo [3], however, whether the DNA-binding is sequence specific is not known. The SWIRM domain of MYSM1, in contrast, does not have direct DNA binding activity [4], and although it is a common domain-type for chromatin-associated proteins [4,5], it was shown to be dispensable for MYSM1 interactions with histones, at least in some systems [3]. Importantly, the MPN metalloprotease domain of MYSM1 is the catalytic domain, characterized by Zn2^+^-binding, the JAMM motif with consensus sequence EXnHSHX_7_SX_2_D [6], and deubiquitinase catalytic activity (DUB) [6].

Our knowledge of the physiological substrates of MYSM1 DUB-catalytic activity continues to expand. Histone H2A, monoubiquitinated at K119, was the first substrate of MYSM1 to be described (H2AK119ub) [7]. More recently MYSM1 was also demonstrated to cleave K63-polyubiquitin chains on cytosolic substrates TRAF3, TRAF6, and RIP2 [3,8]. MYSM1 catalytic activity against polyubiquitin chains of different geometries [9] was subsequently tested using in vitro assays, and MYSM1 was shown to cleave M1, K6, and K27 chains, but was catalytically inactive against K11, K29, K33, and K48 chains [8]. This suggests that our knowledge of MYSM1 substrates remains incomplete and further targets of its catalytic activity may be discovered in the future.

Despite some recent advances, the precise role of MYSM1 in shaping the epigenetic landscape of different mammalian cells remains poorly understood. Histone H2AK119ub is a repressive epigenetic mark deposited on chromatin primarily by polycomb repressive complex 1 (PRC1), for long-term gene silencing during cell differentiation and lineage specification [10,11,12]. In contrast, histone H2BK120ub is an epigenetic mark of transcriptionally active genes [13,14]. The catalytic activity of MYSM1 against histone H2AK119ub, but not H2BK120ub, therefore indicates its primary role as an activator of gene expression [7]. However, MYSM1 is one of many DUBs in mammalian cells that can catalyze H2AK119ub deubiquitination [15]. Other major DUBs with specificity for histone H2AK119ub over H2BK120ub are BAP1 and USP16, while USP3, USP12, USP22, and USP44 deubiquitinate both H2AK119ub and H2BK120ub, as well as different non-histone substrates [15]. How MYSM1 cooperates with the other DUBs to regulate the genome-wide landscape of histone H2A ubiquitination and the gene expression profiles of different mammalian cell types remains poorly understood. Furthermore, MYSM1 activities against polyubiquitinated forms of histone H2A at DNA damage foci [15,16] or against other less well-characterized ubiquitinated histones and histone variants [11,17,18] to our knowledge have not been investigated.

### 1.2. MYSM1-Deficiency in Human and Mouse: Mechanistic Insights and Biomedical Significance

Characterization of *Mysm1*-deficient mouse strains has been instrumental in revealing the essential functions of MYSM1 in different aspects of mammalian physiology [19,20]. *Mysm1*^-/-^ mice exhibit partial embryonic lethality, growth retardation, skeletal and coat pigmentation defects, and complex hematopoietic and immune phenotypes [19], while no phenotypic abnormalities are reported in *Mysm1*^+/-^ heterozygous mice. The International Mouse Phenotyping Consortium (IMPC) [21,22,23] provides extensive primary data on these phenotypes [24]. This and the far-reaching data from recent publications will be covered in depth in subsequent sections of this review.

*MYSM1* mutations in patients with an inherited bone marrow failure syndrome (IBMFS) highlight the biomedical importance of understanding MYSM1 activities and functions. Five patients with homozygous *MYSM1* mutations were characterized in depth [25,26,27], carrying either a p.H656R substitution within the critical JAMM-motif of the catalytic domain, or a nonsense variant p.E390* truncating MYSM1 protein upstream of the catalytic domain [25,26,27] (Figure 1). The variants were therefore predicted to severely impact or fully abolish MYSM1 catalytic activity. All five patients exhibit anemia and leukopenia [25,26,27], in some cases associated with growth retardation [26,27], developmental malformations [27], and neurodevelopmental delay [27]. B cells were severely depleted in all patients [25,26,27], correlating with reduced serum IgM levels [25,26,27], although IgG levels were reduced in only one of the patients [26]. Most patients also showed a reduction in NK cell numbers, while neutropenia was noted in three patients, and T cell depletion was observed in two patients [25,26,27]. Skeletal and craniofacial abnormalities reported in two patients included limb shortening (rhizomelia) and midface hypoplasia [27]. Additionally, a sixth patient also carrying the p.E390* *MYSM1* mutation in a homozygous state was reported to have neutrophilic panniculitis, as well as reduced B cell count, anemia, and a mild growth retardation [28]. Finally, a novel homozygous mutation p.R478* in *MYSM1* was recently identified via whole-exome sequencing in a patient diagnosed with Diamond-Blackfan anemia, a disorder characterized by anemia and to a lesser extent other hematological and developmental abnormalities [29].

Multilineage defects in hematopoiesis in the *MYSM1*-deficient patients suggest impaired hematopoietic stem cell (HSC) function, and this was further supported by several lines of evidence. Thus, a reduction in the frequency of CD34^+^ hematopoietic stem and progenitor cells was reported in the patient homozygous for the *MYSM1*:c.1967A>G:p.H656R mutation [26]. Remarkably, this patient experienced a spontaneous genetic reversion, restoring the normal sequence of one *MYSM1*-allele in hematopoietic cells and resulting in a correction of all immunohematological defects [26]. Assuming that the reversion mutation originated in a single HSC, we can conclude that restoration of MYSM1 function provided a very strong selective advantage and allowed one HSC clone to reconstitute normal wild-type hematopoiesis in competition with a large pool of MYSM1-deficient stem and progenitor cells. This attests to the true importance of MYSM1 for normal HSC function in human [26]. Consistent with this, immunohematological defects in several other *MYSM1*-syndrome patients were successfully cured via allogeneic hematopoietic stem cell transplantation (HSCT) [27].

The high MYSM1 protein homology between human and mouse (87%) and the similarities in the phenotypes of *MYSM1*-deficiency between the species indicate that studies of MYSM1 activities and functions in mouse models can provide important insights into the etiology of *MYSM1*-deficiency syndrome in human. The emerging data from many murine studies published over the past 8 years will be reviewed below.

## 2. Molecular Functions of MYSM1 Protein

### 2.1. MYSM1 Is a Transcriptional Regulator in Hematopoietic Lineage Specification

**Figure 2:**
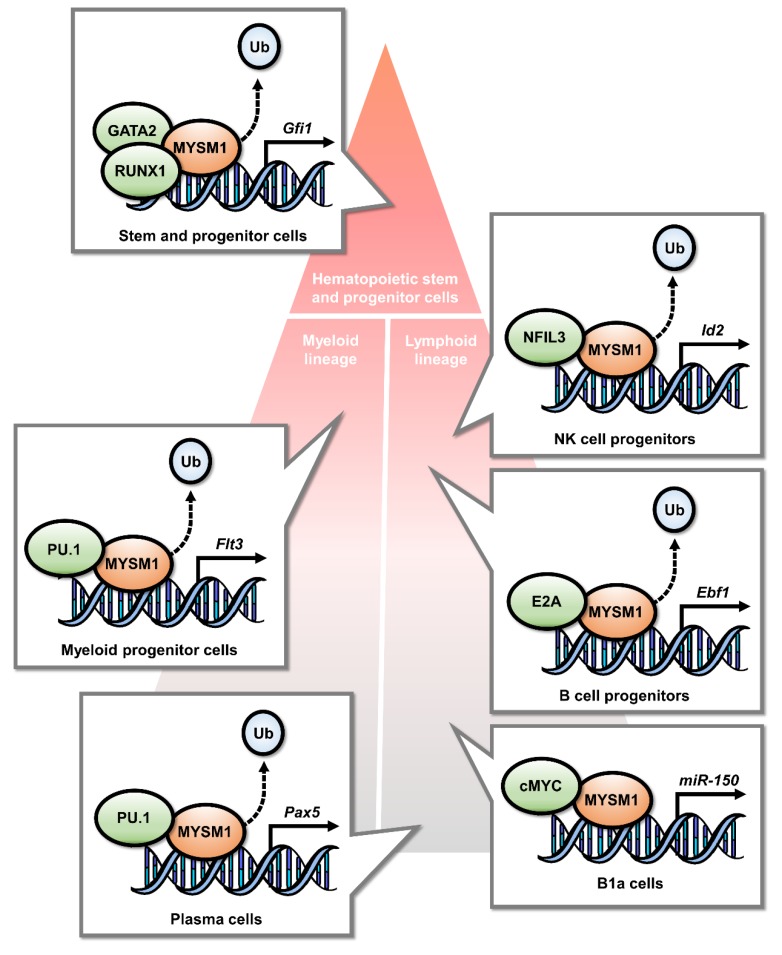
Overview of the reported roles of MYSM1 in the transcriptional regulation of hematopoiesis. MYSM1 was shown to de-repress the expression of *Ebf1* in B cell progenitors [20], *Pax5* in naïve B cells [30], *miR150* in B1a cells [31], *Id2* in NK cell progenitors [32], *Flt3* in dendritic cell precursors [33], and *Gfi1* in hematopoietic stem and progenitor cells [34], through deubiquitination of histone H2AK119ub, and interactions with hematopoietic transcription factors E2A [20], PU.1 [30,33], GATA2 [34], RUNX1 [34], cMYC [31], and NFIL3 [32].

MYSM1 was originally characterized as an epigenetic regulator that promotes the expression of androgen receptor target genes in prostate cancer cell lines, through deubiquitination of histone H2AK119ub [7]. With the discovery of major hematopoietic dysfunction in *Mysm1*-deficient mouse models, most subsequent studies of MYSM1 in transcriptional regulation have focused on hematopoietic cells. These studies elucidated the role of MYSM1 in the de-repression of a range of genes essential for normal stem cell differentiation and lineage specification in hematopoiesis. The genes de-repressed by MYSM1 include: *Ebf1* in B cell progenitors [20], *Pax5* in naïve B cells [30], *miR150* in B1a cells [31], *Id2* in NK cell progenitors [32], *Flt3* in dendritic cell precursors [33], and *Gfi1* in hematopoietic stem and progenitor cells [34] (Figure 2). At many of these loci, loss of MYSM1 resulted in increased binding of PRC1 complex proteins and elevated levels of histone H2AK119ub [20,30,32,33,34], suggesting that MYSM1 antagonizes PRC1-mediated histone ubiquitination and transcriptional repression. Increased levels of histone H3K27me3 were also observed at several loci [20,30,33,34], suggesting indirect cross-talk between MYSM1-deficiency and PRC2 complex activity. Regrettably, only a few studies specifically tested whether the catalytic activity of MYSM1 is essential for its transcriptional activity [7].

MYSM1 was also shown to interact with many transcriptional regulators through co-immunoprecipitation assays, including essential hematopoietic transcription factors E2A [20], PU.1 [30,33], GATA2 [34], RUNX1 [34], cMYC [31], and NFIL3 [32], as well as the histone acetylase pCAF [7], and the BRG1 and BRM catalytic components of the chromatin remodeling complex SWI/SNF [20] (Figure 2). In these studies, the loss of MYSM1 was commonly associated with reduced recruitment of these transcription factors to specific gene promoters and repression of target gene expression [7,20,30,31,32,33,34]. In contrast, pCAF was suggested to act upstream of MYSM1, as MYSM1 catalytic activity was enhanced on hyperacetylated chromatin but inhibited with pCAF-knockdown [7]. However, many unanswered questions remain about the structural basis and functional significance of these MYSM1 protein interactions. Although the interactions were validated through co-immunoprecipitation [7,20,30,31,32,33,34], it is unclear whether the binding is direct and its structural basis remains unknown. It is also unclear whether the binding of MYSM1 to the transcription factors is needed for their enhanced recruitment to specific MYSM1-regulated genes, or whether MYSM1 enhances their recruitment indirectly through chromatin opening and remodeling. Finally, the above studies are restricted to a small number of MYSM1 regulated loci, and the relevance of the proposed mechanisms to MYSM1-transcriptional regulation on a genome-wide scale remains unknown.

### 2.2. MYSM1 Is a Regulator of Signal Transduction Pathways in Innate Immunity

**Figure 3:**
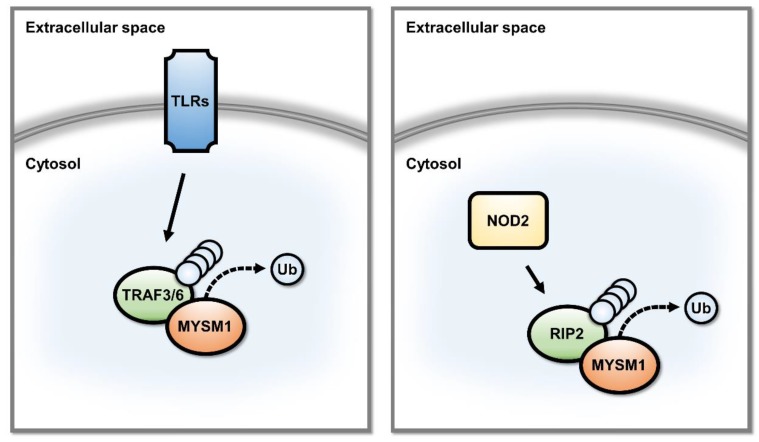
Overview of the cytosolic functions of the MYSM1 protein in the regulation of signal transduction in innate immunity and inflammatory responses in macrophages. MYSM1 was shown to promote deubiquitination of TRAF3, TRAF6, and RIP2 in the signal transduction pathways of TLR and NOD2 pattern recognition receptors, thus, repressing the innate immune and inflammatory responses of macrophages [3,8].

Recent studies indicated an alternative function for MYSM1 as a regulator of the signal transduction pathways of innate immunity in the cytosol, independent of MYSM1-mediated regulation of gene expression at chromatin [3,8]. Thus, a cytosolic pool of MYSM1 protein is produced transiently in mouse macrophages, in response to inflammatory stimulation or infection, involving de novo protein synthesis and subsequent proteasomal degradation [3]. This pool of MYSM1 antagonizes K63-polyubiquitination of TRAF3 and TRAF6 in TLR signal transduction pathways, and K63/K27/M1-polyubiquitination of RIP2 downstream of NOD2 [3,8] (Figure 3). Interestingly, the SWIRM and MPN domains of MYSM1 are required for this activity, but the SANT domain is dispensable [3,8]. Loss of cytosolic MYSM1 function was implicated in the enhanced production of inflammatory cytokine and type-I interferons seen in *Mysm1*^-/-^ macrophages. It also likely accounts for the increased susceptibility to septic shock and peritonitis [3,8], and enhanced clearance of viral infection in mice with systemic or myeloid lineage-restricted *Mysm1*-deletion [3].

Whether MYSM1 has cytosolic functions in other cell types and in regulation of other signaling pathways remains unknown and merits further investigation. Of note, *Mysm1*^-/-^ adipose-derived stem cells were also recently shown to produce higher levels of inflammatory cytokines and to exacerbate the pathology of colitis in mouse models, although MYSM1 regulation of *miR150*-expression was proposed as the underlying mechanism [35,36]. The exact mechanisms mediating MYSM1 retention in the cytosol also remain to be further characterized. Finally, the role of MYSM1 in the regulation of signal transduction in human innate immune response requires further investigation, including the impact on the symptoms and etiology of the human *MYSM1*-deficiency syndrome.

### 2.3. Putative Roles of MYSM1 in DNA Repair

Given the prominent roles of ubiquitination of histone H2A and other proteins in DNA repair [15,16], links between MYSM1 and DNA repair were suggested in several studies [37]. A proteomic screen identified MYSM1 among a large set of putative substrates phosphorylated by ATM and ATR in response to irradiation induced DNA damage [38]. A comprehensive screen of DUB proteins further demonstrated MYSM1 localization to DNA damage foci, and showed that *Mysm1*-knockdown in cell lines triggers spontaneous accumulation of DNA double strand breaks (DSB) and results in impaired repair of induced DNA damage [39]. Increased γH2AX levels were also seen in hematopoietic cells from *MYSM1*-deficiency syndrome patients [27], suggesting increased levels of DNA damage. Furthermore, *Mysm1*-deficient mice were hypersensitive to whole-body ionizing radiation [34] and accumulated increased levels of DNA damage in UV-treated skin [40]. We also observed spontaneous accrual of γH2AX in *Mysm1*-deficient mouse hematopoietic stem and progenitor cells [19], however, this was alleviated in *Mysm1*^-/-^*p53*^-/-^ and *Mysm1*^-/-^*Puma*^-/-^ double-knockout mouse strains [41], suggesting that it was a result of elevated cell apoptosis, rather than of a DNA repair deficiency. To our knowledge, MYSM1 effects on K63-polyubiquitination of histone H2A or other proteins involved in DNA repair or DNA damage response have not been reported. Although BRCC36 is known to be the major DUB with activity on polyubiquitinated histone H2A at DNA damage foci [15,16], a possible role for MYSM1 does merit further investigation.

### 2.4. MYSM1 and the p53 Stress Response Pathway

p53 is a transcription factor commonly called the “guardian of the genome” due to its central role in regulating cellular responses to stress, including cell cycle arrest, senescence, apoptosis, DNA repair, and autophagy [42,43]. Although p53 activation was classically studied in the context of DNA damage and oncogene activation, a variety of cellular stresses are now known to trigger its activity.

Several studies noted an increase in p53 protein levels and activation of p53-regulated stress response genes in *Mysm1*-deficient mouse hematopoietic stem and progenitor cells [19,44,45]. This was also observed after an inducible deletion of *Mysm1* in adult mice, indicating that MYSM1 is required constitutively to repress p53-activation [46].

Importantly, the silencing of p53 stress response in *Mysm1*^-/-^*p53*^-/-^ double-knockout mice resulted in a visible rescue of *Mysm1*^-/-^ phenotype, restoring mouse body size, normal morphology of the hind-limbs, tail and skin [44,45,47,48], the cellularity of the blood and lymphoid organs, and the numbers of hematopoietic stem and progenitor cells in the mouse bone marrow [44,45]. *Mysm1*^-/-^*p53*^-/-^ HSCs were fully capable of reconstituting hematopoiesis following transplantation, indicating restoration of normal HSC functions [44]. Altogether, these studies indicated that p53 activation is the common mechanism driving hematopoietic dysfunction and other phenotypic abnormalities in *Mysm1*-deficiency.

Further characterization of the mouse strains deficient for the important mediators of p53-dependent apoptosis *Bbc3*/PUMA and cell cycle arrest *Cdkn1a*/p21 elucidated the specific pathways downstream of p53 mediating the *Mysm1*-deficiency phenotype. In particular, these studies demonstrated a partial rescue of *Mysm1*-phenotypes in *Mysm1*^-/-^*Bbc3*^-/-^ but not in *Mysm1*^-/-^*Cdkn1a*^-/-^ animals [41]. The studies therefore concluded that the p53-driven over-expression of *Bbc3*/PUMA drives the apoptosis of hematopoietic multipotent progenitor cells (MPPs) and the depletion myeloid lineage cells, but not the arrest in lymphocyte development in *Mysm1*-deficiency [41].

The molecular mechanisms connecting *Mysm1*-deficiency and p53-activation remain somewhat controversial. Interaction between MYSM1 and p53 proteins was demonstrated in hematopoietic cell lines through co-immunoprecipitation (co-IP) [41], and MYSM1 binding to the promoters of several p53 stress response genes was also reported, suggesting possible mechanisms for p53 regulation by MYSM1. Thus, in thymocytes, MYSM1 binds at the *Cdkn2a* locus that encodes upstream regulators of p53 activation p16/INK4A and p19/ARF, and the expression and protein levels p19/ARF are elevated in *Mysm1*-deficient thymocytes [45]. In pro-B cell lines, MYSM1 binds at the promoters of p53-regulated genes *Bbc3*/PUMA and *Cdkn1a*/p21 that encode important mediators of p53-dependent apoptosis and cell cycle arrest, respectively [41]. MYSM1-binding sites at these promoters coincide with known p53-binding sites, and *Mysm1*-knockdown results in increased p53 binding, increased levels of activating histone marks H3K27ac and H3K4me3, and increased gene expression [41], suggesting that MYSM1 is a negative regulator of these loci. It is important to note however that these experiments were limited to a small set of putative MYSM1-regulated loci [41,45], and thus do not rule out existence of other mechanistic links between *Mysm1*-deficiency and p53 activation. The studies were also conducted primarily in lymphoid precursor cells [41,45], while a conditional deletion of *Mysm1* in these cells in vivo has minimal impact on lymphocyte development [49,50], stressing the importance of conducting further mechanistic studies on the cross-talk between MYSM1 and p53 in earlier hematopoietic precursors and HSCs. 

## 3. MYSM1 Regulated Checkpoints in Mammalian Physiology

### 3.1. Essential and Cell Intrinsic Functions of MYSM1 in Hematopoietic Stem Cells

Many lines of evidence support the essential role of MYSM1 in hematopoiesis and HSCs. Thus, multilineage defects in hematopoiesis are a common feature of *Mysm1*-deficiency in human patients and mouse models, with *Mysm1*^-/-^ mice exhibiting a severe depletion of B cells and NK cells, and a milder reduction in T cells, myeloid leukocytes, and erythroid lineage cells [19]. Importantly, MYSM1 regulates hematopoiesis through cell intrinsic mechanisms, as demonstrated by its expression in HSCs and other hematopoietic cells [34], mouse-to-mouse bone marrow transplantation experiments [19,34], and the selective deletions of *Mysm1* within hematopoietic cells in Cre/loxP mouse models [51,52]. Further mechanistic studies in mice established that *Mysm1*-deficiency preserves HSC numbers, as defined by the cell surface markers Lin^-^cKit^+^Sca1^+^CD150^+^CD48^-^CD34^-^Flt3^-^, however, these cells exhibit loss quiescence and fail to engraft hematopoiesis following transplantation, indicating loss of HSC function [19,34]. The loss of HSC function is observed in both *Mysm1*^-/-^ fetal liver and adult bone marrow [46], indicating the importance of MYSM1 in hematopoiesis through multiple stages of ontogenesis. Its role specifically in the emergence of HSCs in embryogenesis to our knowledge has not been specifically investigated. Importantly, data from human patients also supports the cell-intrinsic role of MYSM1 in HSC function, as shown by the correction of all immunohematological defects in one of the patients following a spontaneous genetic reversion of the *MYSM1*:p.H656R mutation in the patient’s hematopoietic cells [26].

At the molecular level, MYSM1 was shown to regulate HSC function in part through the induction of the gene encoding transcription factor GFI1 [34]. Indeed, *Gfi1*-expression was reduced in *Mysm1*^-/-^ HSCs, and MYSM1 was shown to bind to *Gfi1* promoter in lineage-negative bone marrow cells [34]. MYSM1 was required for normal recruitment of transcription factors GATA2 and RUNX1 to the *Gfi1* locus, while loss of MYSM1 resulted in increased recruitment of the PRC1 complex proteins RING1B and BMI1 and increased levels of histone H2AK119ub [34] (Figure 2). Furthermore, retroviral expression of *Gfi1* in *Mysm1*^-/-^ cells could partly restore HSC quiescence and function [34]. Despite strong mechanistic evidence, it is important to note that these studies did not conduct genome-wide analyses of MYSM1-regulated genes in HSCs, and therefore cannot rule out possible alternative mechanisms for MYSM1 regulation of HSC function. Indeed a number of recent studies reported activation of p53 stress response in *Mysm1*^-/-^ hematopoietic stem and progenitor cells (HSPCs) and a rescue of hematopoiesis and other *Mysm1*-deficiency phenotypes in *Mysm1*^-/-^p53^-/-^ double-knockout mice [44,45]. Although a mechanistic link between *Gfi1*-loss and p53 activation was previously reported in other models [53,54], it is not clear at this point how p53 activation and the reduction in *Gfi1* expression are mechanistically connected in the *Mysm1*^-/-^ mouse model.

### 3.2. MYSM1 in B Cell Development and Humoral Immune Response

Depletion of the B cell lineage is one of the major common features of *MYSM1*-deficiency in human patients and mouse models. The arrest in early B cell development in *Mysm1*-deficient mice was linked to the MYSM1 role in the induction of the expression of *Ebf1* gene, encoding an essential transcription factor for B cell lineage specification [20]. MYSM1 binding was detected at the *Ebf1*-locus promoter in B cells, *Ebf1*-expression was reduced in *Mysm1*-deficient pre-pro-B cells, and retroviral expression of *Ebf1* in *Mysm1*-deficient bone marrow could partly rescue B cell development in an in vitro assay [20]. Reduced expression of *Ebf1* in *Mysm1*-deficiency correlated with reduced recruitment of transcription factor E2A, increased recruitment of PRC1 complex proteins, and increased levels of histone H2AK119ub at the *Ebf1*-locus [20] (Figure 2). An interaction of MYSM1 with the BRM and BRG1 components of the SWI/SNF chromatin remodeling complex was also suggested to have a role in the regulation of *Ebf1* locus expression [20].

Surprisingly, despite the severe depletion of B cells, *Mysm1*^-/-^ mice had normal antibody levels, could mount normal antigen-specific antibody responses to immunization, and had increased frequencies of antigen-specific plasma cells in their lymphoid organs, although the absolute numbers of plasma cells were nevertheless reduced [30]. Furthermore, *Mysm1*^-/-^ plasma cells showed enhanced levels of antibody secretion in vitro and had altered gene expression profiles with downregulation of B cell lineage transcription factors *Pax5* and *Bach2*, and increased expression of plasma cell transcription factors *Blimp1* and *Xbp1* [30]. Altogether, MYSM1 was proposed to inhibit plasma cell differentiation by maintaining *Pax5* expression, via histone H2AK119-deubiquitination and recruitment of transcription factor PU.1 to the *Pax5* locus [30] (Figure 2). In another study, MYSM1 was also reported to act as a negative regulator of B1a cell development or expansion, by maintaining the expression of microRNA miR-150, working in concert with transcription factor cMYC [31] (Figure 2). This microRNA is a known regulator of hematopoiesis and lymphocyte development, modulating the expression of multiple targets through post-transcriptional mechanisms, such as repression of translation and promoting the degradation of specific mRNAs [36]. It is important to note however that although B1a cells represented a larger fraction of the overall B cell population in *Mysm1*^-/-^ mice, they were nevertheless strongly reduced in absolute numbers in this animal model [31].

To characterize the checkpoints in lymphocyte development and adaptive immune response regulated by MYSM1, independently of MYSM1 functions at the earlier stages of hematopoiesis, we performed a deletion of *Mysm1* at different stages of B and T cell development in Cre/loxP mouse models [49,50]. Surprisingly, *Mysm1*-deletion from the pro-B cell stage (mb1-Cre) resulted in a mild depletion of B cell numbers, while *Mysm1*-deletion at the pre-B cell stage (CD19-cre) or in mature follicular B cells (CD21-cre) allowed the normal numbers of B cells to be maintained [50]. These B cells also retained normal levels of *Ebf1* and *Pax5* gene expression, despite a full loss of *Mysm1* transcript [50]. This indicates that the severe loss of B cells in *Mysm1*^-/-^ mice is primarily due to upstream defects in lymphoid lineage specification, and not due to the cell-intrinsic functions of MYSM1 in B cells. Intriguingly, mature splenic B cells from *Mysm1*^fl/fl^ mb1-cre mice had altered responses to in vitro stimulation, including increased expression of activation markers, impaired survival, and reduced proliferation, while *Mysm1*^fl/fl^ CD21-cre B cells showed no such defects. This suggests that MYSM1 activity at early stages of B cell development may be required for epigenetic programing to allow normal responses to stimulation and engagement in immune response in mature B cells. Additionally, *Mysm1*^fl/fl^ mb1-cre mice had normal serum antibody levels, normal antigen-specific antibody titres in response to immunization, and showed a trend toward an increase in plasma cell numbers, despite a reduction in overall B cell numbers [50], providing some support for the negative role of MYSM1 in the regulation of plasma cell differentiation [30].

Overall, the above studies indicated that MYSM1 plays a critical role in the early stages of B cell lineage specification and possibly also to a lesser extent in the regulation of plasma cell development in B-cell-mediated immune response. The exact mechanisms for B cell lineage depletion discussed above remain to be reconciled with the apparent role of p53 and the rescue of B cell development seen in *Mysm1*^-/-^*p53*^-/-^ mice [19,44,45]. Furthermore, the striking differences in the B cell phenotypes of mice with systemic versus conditional *Mysm1*-deletion highlight the importance of understanding the specific functions of MYSM1 at different stages in B cell lineage development, and the possible mechanisms of epigenetic memory, through which MYSM1 activity at early stages of development may affect B cell functions later in their maturation history.

### 3.3. MYSM1 in T Cell and NK Cell Development

Several studies explored the defects in T cell and NK cell differentiation in *Mysm1*-deficient mouse models. Thus *Mysm1*^-/-^ mice have a severe reduction in mature NK cell numbers in the bone marrow, blood, and lymphoid organs [32]. This defect in NK cell maturation is attributed to the cell intrinsic role of MYSM1 in the induction of the gene encoding transcription factor ID2, known to be important for the NK cell lineage [32]. MYSM1 binding was detected at the *Id2*-locus promoter and loss of MYSM1 was associated with reduced recruitment of transcription factor NFIL3, increased recruitment of PRC1 complex proteins, increased levels of histone H2AK119ub, and reduced gene expression [32] (Figure 2). To our knowledge, the role of p53 activation in *Mysm1*-deficient NK cell dysfunction has not been addressed [19,44,45].

T cells are also significantly reduced in absolute numbers in the peripheral lymphoid organs of *Mysm1*^-/-^ mice [19,45,55], and this is associated with a severe reduction in the cellularity of the thymus and a depletion of all thymocyte subsets [19,45,55], including the early thymic progenitors (ETPs) [45]. The underlying cellular mechanisms likely include the upstream defects in lymphoid lineage specification within the bone marrow [19,45,55], as well as elevated levels of cell apoptosis within the thymus [45]. At the molecular level, activation of the p53-stress response was extensively characterized and shown to be functionally important, based on the rescue of T cell development in *Mysm1*^-/-^*p53*^-/-^ mice [44,45]. The suggested triggers for p53 activation in *Mysm1*^-/-^ include the putative roles of MYSM1 in the maintenance of p19ARF expression within the thymus [45], and IRF2 and IRF8 expression in bone marrow progenitor cells [55]. 

To characterize MYSM1 functions within the T cell lineage, independently of its roles at earlier stages of hematopoiesis, we performed a conditional deletion of *Mysm1* in Cre/loxP mouse models [49]. Deletion of *Mysm1* in the thymus from the double-negative 3 (DN3, Lck-Cre) or the double-positive (DP, CD4-Cre) stage in T cell lineage development resulted in normal thymocyte numbers and normal progression of thymic T cell development [49]. In peripheral lymphoid organs, mild depletion of CD8 T cells and elevated expression of activation markers on both CD8 and CD4 T cells were observed [49]. T cell responses to in vitro stimulation were also altered, with CD8 T cells showing elevated cytokine production, reduced induction of granzyme B, impaired proliferation, and increased apoptosis, while CD4 T cells showed only impaired proliferation [49]. Altogether, this indicated that the defects in T cell development in *Mysm1*-deficiency are primarily the result of upstream defects in lymphoid lineage specification within the bone marrow, but suggested some role for MYSM1 in the regulation of mature CD8 T cell activation, proliferation, and survival. Importantly, increased levels of p53 were observed in CD8 T cells from the *Mysm1*^fl/fl^ CD4-cre mice [49], suggesting that the mechanisms for CD8 T cell dysfunction in this model might also be p53-dependent.

### 3.4. MYSM1 in Myeloid Lineage Cell Development and Innate Immune Response

The development of myeloid lineage leukocytes is also significantly impaired in *Mysm1*^-/-^ mice, as demonstrated by the reduction in the absolute numbers of granulocytes, monocytes, and macrophages in peripheral lymphoid organs, and their progenitors in the bone marrow [33]. A profound requirement for MYSM1 in the development of dendritic cells (DCs), including both conventional and plasmacytoid lineages, was also reported [33]. Expression of receptor tyrosine kinase *Flt3*, known to be essential for DC development, was reduced in *Mysm1*^-/-^ hematopoietic progenitors, and exogenous expression of *Flt3* through retroviral transduction in these cells could partially rescue DC development in vitro [33]. MYSM1 bound to the *Flt3* gene promoter, and loss of MYSM1 resulted in reduced recruitment of the major hematopoietic transcription factor PU.1 and an increase in repressive histone marks H2AK119ub and H3K27me3 at the *Flt3* locus [33] (Figure 2).

MYSM1 was also shown to have a direct role in the regulation of macrophage activation, in response to inflammatory stimuli and infection. Importantly, these MYSM1 activities were linked not to MYSM1 functions as a transcriptional regulator at chromatin, but relied on a cytosolic pool of MYSM1 protein that was transiently produced in activated macrophages [3,8]. This pool of MYSM1 worked to remove polyubiquitin chains from TRAF3, TRAF6, and RIP2, thereby, silencing TLR and NOD2 signal transduction pathways [3,8] (Figure 3). *Mysm1*^-/-^ macrophages therefore showed enhanced production of inflammatory cytokines and type-I interferons, and mice with either systemic or myeloid lineage-restricted *Mysm1*-deletion were more susceptible to septic shock and peritonitis [3,8], but showed enhanced clearance of viral infections [3]. In a separate study, *Mysm1*^-/-^ macrophages were confirmed to have elevated production of pro-inflammatory cytokines in response to stimulation, as well as increased proliferation, increased apoptosis, and enhanced capacity to control melanoma tumor growth in vivo in mouse models [56].

### 3.5. MYSM1 Functions beyond the Hematopoietic System

MYSM1 functions beyond the hematopoietic and immune system are apparent from visual examination of *Mysm1*-deficient mouse strains, which exhibit growth retardation, dysmorphology of hind-limbs and tail, and abnormal coat pigmentation (white belly patch) [19]. The most comprehensive source of information on the phenotypes of *Mysm1*-deficiency is provided by the International Mouse Phenotyping Consortium (IMPC) [21,22,23], and this catalogues other skeletal, metabolic, neurological, and behavioral phenotypes. Despite the comprehensive characterization of *Mysm1*^-/-^ phenotypes by IMPC, only a few systems beyond hematopoiesis and immunity have been explored at a mechanistic level [24]. For example, skin phenotypes of *Mysm1*^-/-^ mice were further characterized in several studies, reporting reduced cellularity and altered morphology of interfollicular epidermis, abnormal patterning of hair follicles and sebaceous glands, and disruptions of the hair follicle cycle [47,57,58]. This was associated with depletion of epidermal stem cells, reduced colony formation by epidermal progenitors, and activation of the transcriptional signatures of p53 stress response in the skin samples [47]. Abnormalities in the in vitro differentiation of *Mysm1*^-/-^ mesenchymal stem cells (MSCs), such as enhanced adipogenesis, were also reported, and could be functionally linked to osteopenia and skeletal abnormalities in *Mysm1*-deficiency [48].

### 3.6. Possible Roles of MYSM1 in Cancer

Several recent studies explored the role of MYSM1 in various cancer models and suggested both pro- and anti-carcinogenic functions of MYSM1 protein. *Mysm1*-deficiency is known to result in p53-activation in hematopoietic cells [19,44,45], indicating that MYSM1 normally represses p53 activation, and suggesting it as a possible target for therapeutic p53-activation in hematological malignancies that retain wild type p53 function. At the same time, *Mysm1*-deficient mice are known to develop spontaneous thymic lymphomas at 6–9 months of age [44]. These tumors have not been extensively characterized, may carry genetic changes that silence the p53 stress response pathway, and may arise through mechanisms common with other mouse strains with impaired production of thymic progenitors [59]. Several other studies also suggested a role for MYSM1 as a positive regulator of cancer progression. Thus, MYSM1 was originally characterized as a positive regulator of androgen receptor target gene expression in human prostate cancer cell lines [7]. Furthermore, elevated MYSM1 protein levels were observed in human melanomas compared to normal melanocytes, and *Mysm1*-knockdown impaired the proliferation and survival of melanoma cell lines [40]. Moreover, increased MYSM1 protein levels were also seen in human colorectal tumors, compared to adjacent normal mucosa [60]. No malignancies have been reported to date in *MYSM1*-deficiency syndrome in human [25,26,27], however, due to the low patient numbers and their young age, our understanding of cancer susceptibility in human *MYSM1*-deficiency may be incomplete.

## 4. Conclusions

The discovery of *MYSM1* mutations in patients with a hereditary developmental and bone marrow failure syndrome raises strong interest in understanding the MYSM1-regulated checkpoints in mammalian physiology and the underlying molecular mechanisms of MYSM1 activity in these systems. Over the past decade, research in mouse models has provided numerous important insights, although many unanswered questions and opportunities for discoveries remain. The role of MYSM1 as a transcriptional regulator of hematopoiesis and immune cell development has been most extensively studied, although the diverse findings remain to be consolidated and reconciled. In particular, genome-wide characterization of MYSM1-regulated genes in the relevant hematopoietic cell types promise to provide many new insights into MYSM1 functions. The recently discovered role of MYSM1 in the regulation of signal transduction in the cytosol is highly interesting, however, research to date has focused only on the signaling pathways of innate immunity and primarily in macrophages. Furthermore, except for several recent insightful studies, MYSM1 functions and mechanisms of action beyond hematopoiesis and immunity remain poorly characterized. We expect that many new studies in coming years will address these and other outstanding questions, providing insights into the basic molecular mechanisms regulating mammalian development and physiology and further understanding into the disease pathology in *MYSM1*-deficiency in humans.
